# Molecular Foundations of Neuroplasticity in Brain Tumours: From Microscopic Adaptation to Functional Reorganisation

**DOI:** 10.3390/ijms26157049

**Published:** 2025-07-22

**Authors:** Lizeth Vinueza, Salvador Pineda, Jose E. Leon-Rojas

**Affiliations:** 1 Cerebro, Emoción y Conducta (CEC) Research Group, Escuela de Medicina, Universidad de las Américas (UDLA), Quito 170124, Ecuador; lizeth.vinueza.mera@udla.edu.ec; 2 NeurALL Research Group, Quito 170157, Ecuador; salpigue@gmail.com

**Keywords:** neuroplasticity, brain tumours, molecular mechanisms, glioma surgery, functional reorganisation

## Abstract

Brain tumours challenge the structural and functional integrity of the brain, yet remarkable neuroplastic adaptations often preserve critical functions. This review synthesises the current knowledge of the molecular events underlying neuroplasticity in the context of tumoural growth, spanning from early genetic and protein alterations to macroscopic functional reorganisation. We discuss the roles of stress-regulated molecules, synaptic proteins, trophic factors, and morphological changes in driving adaptive responses. Furthermore, we bridge the gap between microscopic molecular events and large-scale network adaptations, emphasising clinical implications for glioma surgery and patient outcomes. Despite advances, knowledge gaps persist regarding the dynamics, predictors, and therapeutic modulation of plasticity, underscoring the need for future longitudinal and translational research. Understanding and leveraging these molecular mechanisms holds promise for improving functional recovery and quality of life in patients with brain tumours.

## 1. Introduction

The human brain possesses an extraordinary capacity for adaptation, a phenomenon termed neuroplasticity, which is fundamental for functional preservation in the context of structural insults such as neoplastic invasion. In cases where tumours encroach upon or destroy functional tissue, the brain’s intrinsic plastic mechanisms enable it to reorganise and, to varying extents, maintain operational capacity [1]. This adaptive potential is thought to originate primarily at the molecular level, where early biochemical changes set the stage for macroscopic functional and morphological rearrangements [1,2]. Despite an increasing body of literature describing tumour-related plasticity, the precise molecular pathways and the interrelations between micromolecular alterations and larger-scale cortical remodelling are still an evolving area of study.

**Figure 1:**
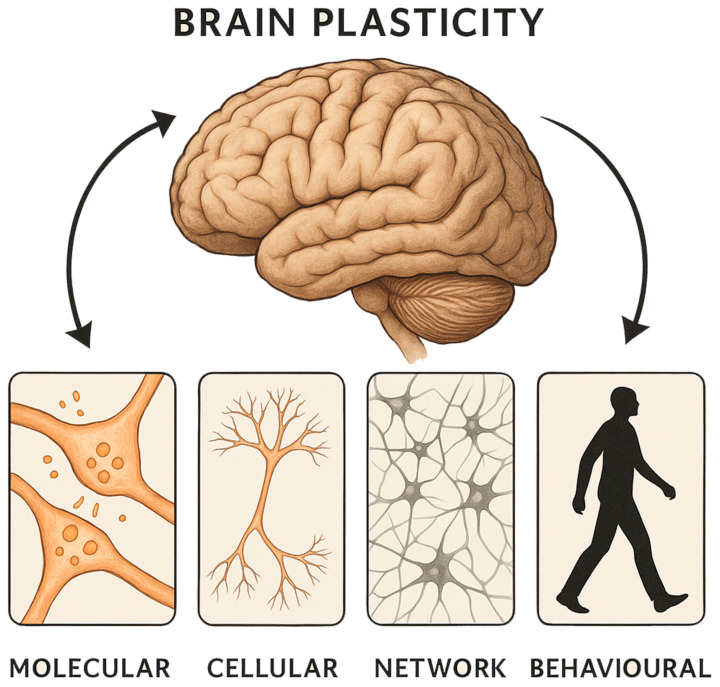
A summary of the main neuroplastic mechanisms associated with brain tumours.

At the microscopic level, the brain’s response to tumour invasion involves a complex and dynamic interplay between genes, proteins, and signalling molecules (Figure 1). Multiple adaptive mechanisms have been proposed, including the modulation of synaptic efficacy, the unmasking of pre-existing but functionally silent connections, phenotypic shifts in neuronal populations, and adult neurogenesis [3,4]. These changes suggest a fundamental plastic substrate that, although originally characterised in models of injury and neurodevelopment, appears to be recruited in the oncological setting as well [3,5]. Particularly, alterations in neurotransmitter receptor expression, synaptic vesicle dynamics, and intracellular signalling pathways have been implicated in this process [6,7]. Beyond the microscopic scale, macroscopic mechanisms of plasticity have also been documented. Diaschisis, whereby remote but functionally connected regions exhibit suppressed activity following a focal insult, can paradoxically facilitate recovery through the reorganisation of functional networks [8]. Redundancy within brain networks, particularly in systems subserving critical functions such as language and motor control, provides an anatomical substrate for functional compensation [5,9,10]. Cross-modal plasticity, whereby brain regions not primarily involved in the compromised function are recruited to assume novel roles, represents another important mechanism; this phenomenon has been well-documented in sensory substitution following deafferentation and is increasingly recognised in the context of tumour-related cortical remapping [11,12]. Furthermore, morphological changes such as axonal sprouting, dendritic arborisation, and volumetric modifications of grey and white matter structures have been identified, suggesting that plasticity is not merely functional, but structural at multiple levels [2,13].

Recent advances in neuroimaging, including diffusion tensor imaging (DTI) and functional MRI (fMRI), have allowed unprecedented insights into these plastic processes. Studies employing these modalities have demonstrated not only functional shifts, but also microstructural adaptations, reinforcing the notion that plasticity operates across a continuum of spatial scales [14,15]. Nonetheless, considerable knowledge gaps persist regarding the temporal dynamics of these changes, their molecular triggers, and the limits of plastic potential in the context of aggressive tumour progression. Therefore, in this review, we aim to describe the sequence of plasticity events, with a particular emphasis on molecular mechanisms, beginning with the earliest microscopic alterations; by synthesising the current literature, we intend to clarify the biological underpinnings of brain plasticity in the setting of tumoural disease, highlighting not only established findings, but also areas where further research is critically needed.

## 2. Plasticity Events at the Micromolecular Level

Changes in genes, proteins, and molecules represent the principal microscopic events observed in the brain in the context of tumours, epilepsy, and ischaemia [1]. However, few studies have characterised in detail the specific molecular alterations occurring in tumour-infiltrated brain tissue, warranting further exploration. In this review, we first address genetic modifications and subsequently discuss protein and molecular changes.

### 2.1. Genetic Changes

Changes in gene expression represent the earliest microscopic events observed in the brain’s response to tumoural growth. In the context of brain tumours, research has predominantly focused on two groups: neuronal growth-associated molecules and synapse-related proteins. For example, M6a, a stress-regulated glycoprotein, is upregulated following brain lesions and facilitates neurite outgrowth and synapse formation [16]. Similarly, MAP2 (Microtubule-Associated Protein 2), a cytoskeletal protein, has been shown to promote dendritic arborisation and neuronal migration, contributing to the reorganisation of neural circuits [17,18]. Experimental models have also demonstrated that GFAP (Glial Fibrillary Acidic Protein), typically associated with astrocytic structure and function, is upregulated in gliosarcoma, reflecting astrocytic activation and the establishment of a supportive environment for neuronal repair [19]. In contrast, upregulation of molecules such as Major Histocompatibility Complex Class I (MHC I) proteins appears to negatively impact synaptic stability, suggesting that not all genetic changes are conducive to beneficial plasticity [20]. Furthermore, glutamatergic dysregulation, reflected by elevated glutamate levels and altered Glu/GABA ratios, may exacerbate excitotoxicity, thereby impairing plastic responses [21]. This duality highlights the complex nature of neuroplasticity in the tumoural brain: some pathways are protective and compensatory, while others may accentuate injury and dysfunction.

Current evidence, largely derived from rodent models, suggests that the upregulation or downregulation of specific plasticity-associated genes is highly dynamic and context-dependent. However, direct evidence from human glioma models remains sparse, underscoring a critical gap in the field that future investigations must address. In 2002, Keyvani & Schallert suggested the following organization of neuroplastic genetic mechanisms/events: (1) transcription factors, (2) kinase network molecules, (3) neurotransmitter receptors, (4) growth factors/receptors, (5) neuronal growth-associated molecules and cytoskeletal molecules, (6) synapse-related molecules, and (7) adhesion molecules [1]. However, when focusing specifically on neuroplastic changes secondary to brain tumours, we only found research reporting mechanisms related to neuronal growth-associated molecules, cytoskeletal molecules, synapse-related molecules, and adhesion molecules [16,17,18,19,20,21,22,23]. Table 1 summarizes the main molecules and proteins that have been reported to be involved in brain plasticity in the setting of a glioma in rodent models.

### 2.2. Trophic Factors and Protein Changes

Trophic factors and associated proteins are pivotal in orchestrating the brain’s adaptive responses to tumour-induced injury (Table 1). These molecules not only sustain neuronal survival, but also actively promote neurite extension, synaptic plasticity, and the remodelling of neural circuits. Among the key modulators, podoplanin has emerged as a significant regulator of neuroplastic processes [19,24]. Podoplanin, a cell surface glycoprotein, is highly expressed under pathological conditions, including brain tumours, and interacts with nerve growth factor (NGF), a well-established neurotrophin essential for the maintenance and plasticity of hippocampus-dependent memory circuits [19]. Experimental evidence indicates that podoplanin regulates neuronal outgrowth, synaptic density, and memory-related plasticity through activation of p-Ezrin and cAMP response element-binding protein (CREB) pathways [19,24]. These downstream signalling events culminate in enhanced neurogenesis and synaptogenesis, both of which are vital for adaptive responses to localised brain injuries. In addition to podoplanin, studies involving gliosarcoma models have demonstrated the upregulation of other proteins such as glial fibrillary acidic protein (GFAP) and von Willebrand factor (vWF) [17,25,26]. GFAP upregulation reflects astrocyte activation, a hallmark of reactive gliosis, which contributes to the creation of a microenvironment conducive to neural repair [25]. Concurrently, increased vWF levels suggest enhanced angiogenesis, supporting the metabolic demands of reorganising neural tissue [17,26].

Synaptophysin, a critical marker of synaptic density, has also been observed to increase in peri-tumoural regions. Its upregulation implies active synaptogenesis, further emphasising the robust compensatory mechanisms triggered by the presence of tumours [16,20]. Conversely, detrimental changes have been reported, including reductions in neurofilament proteins, tyrosine hydroxylase, and dopamine transporter levels, indicating that tumour growth may simultaneously induce degenerative processes in specific neural populations [23]. Collectively, these trophic and protein-level changes constitute a dynamic and complex response that can either facilitate functional compensation or, in certain contexts, exacerbate neurological decline. Future research should aim to delineate how the balance between these opposing forces may be therapeutically modulated to enhance beneficial neuroplasticity.

### 2.3. Synaptic Changes

Synapses are the means by which neurons communicate microscopically; they are not static nor fixed, but instead constantly undergoing dynamic changes. This characteristic makes them a crucial site for plasticity. Brain tumours, particularly gliomas and gliosarcomas, induce profound synaptic changes within the surrounding neural tissue, driven by both tumoural invasion and the host brain’s neuroplastic responses. Tumour cells disrupt local neuronal circuits through direct synaptic contact, paracrine signalling, and metabolic competition, leading to synaptic loss, dendritic spine remodelling, and impaired synaptic transmission [27]. Furthermore, glioma cells have been shown to establish functional pseudo-synapses with neurons, thereby hijacking synaptic activity to promote tumour growth and invasion [28]. Certainly, active neurons have been shown to promote high-grade glioma proliferation and growth by means of secreting mitogens, such as the synaptic protein neuroligin-3 (NLGN3), a leading candidate mitogen [28]. However, in response, the healthy brain can activate beneficial neuroplastic mechanisms, including increased axonal sprouting, synaptogenesis, and astrocytic remodelling, aimed at preserving function and reorganising neural networks [29]. While neuroplasticity may partially compensate for tumour-induced damage, sustaining cognitive function despite progressive structural disruption, maladaptive plasticity can also enhance epileptogenesis and contribute to cognitive decline [30].

Glial reactivity, characterised by astrocyte activation and the upregulation of proteins such as GFAP, plays a pivotal role in modulating the synaptic environment, influencing both reparative and deleterious outcomes [31]. For instance, in a rodent model, researchers investigated the plasticity events occurring in the peri-tumoural areas after implanting glioma cells in the sensorimotor cortex [17]. Among the analysed molecules were the microtubule-associated protein 2 (MAP2), GFAP, vWF, and synaptophysin, which are markers of neurons, astrocytes, endothelial cells, and synaptogenesis, respectively [17]. MAP2 is a cytoskeletal protein found in the dendritic area of neurons and has an important role in neuronal migration and neurite outgrowth; meanwhile, synaptophysin is a presynaptic vesicle protein that could represent a marker of synaptic density [10,17]. By using immunohistochemical techniques, the authors found alterations in the concentrations of these brain molecules; specifically, the authors found that peri-tumoural areas responded with an increase in MAP2, GFAP, VWF, and synaptophysin compared to control brain areas [17]. This finding suggests that the mentioned molecules might be acting in response to the presence of the tumour and, thus, producing compensatory responses such as axonal growing, presynaptic adaptations, and/or synaptogenesis. Furthermore, the process of angiogenesis occurring in unaffected peri-tumoural tissue may act as an adaptation to hypoxia/ischemia and may contribute to the recovery of brain tumourigenic areas and synaptic plasticity [10,17].

As previously mentioned, healthy synapses are constantly changing, being strengthened or reformed, to enable complex abilities such as learning and memory [32]. The presence of brain tumours can alter these major executive functions. The regulation of N-methyl-D-aspartate (NMDA) and α-amino-3-hydroxy-5-methyl-4-isoxazolepropionic acid (AMPA) receptors due to diverse neural stimulation is called long-term potentiation (LTP) [32]. The main mechanism for normal learning and memory and the ongoing paradigm of neuroplasticity at the microscopic level are thought to be influenced by LTP. This microscopic characterization of plasticity provides a physiological explanation for how synapses are continuously modulated during normal conditions [32]. Neuroplasticity after brain injury is still less understood; nevertheless, it is very clear that larger-scale macroscopic plasticity plays a key role in cerebral recovery and reorganization following injury where synaptic-level changes might presumably play a role in the redistribution of function [33]. A group of researchers, investigating the effects of glioma implantation on synaptic plasticity, used rodent models to record short-term potentiation (STP) and LTP in the CA1 region of the contralateral hippocampus, where the tumour was implanted [21]. Interestingly, they found a significant decrease in the maintenance of STP and LTP compared to control groups. Additionally, they encountered a significant increase in glutamate concentrations, but no change in GABA concentrations, as shown by high-performance liquid chromatography [21]. Such findings may be indicators of reduced plasticity. This is thought to occur due to excitotoxicity, a state where high glutamate concentrations and Glu/GABA ratio changes compromise the synapse [21].

These findings highlight the dual-edged nature of neuroplasticity in the context of brain tumours and synaptic plasticity: while it offers a substrate for functional compensation, it simultaneously contributes to network dysfunction and tumour progression.

### 2.4. Neuronal Morphological/Structural Changes

Brains subjected to stressful events, such as tumoural masses, seek alternative forms to adapt. A specific adaptation mechanism is to change the expression of molecules that alter the morphology and/or structure of neurons; these structural molecules that allow morphological changes can be distributed in strategic locations such as dendrites, axons and neurites, and other relevant regions [16]. Studies on the molecular morphological changes in neurons due to tumour invasion are scarce in the scientific literature. Initially, research was focused on studying cellular changes in response to stress. In a study by Goel et al., the cellular pathology of a brain infiltrated by diffuse astrocytoma was investigated using immunohistochemical methods [18]. Their main objective was to describe the molecular responses in peri-tumoural neurons under stress; they described several structural proteins, but the ones with possible plasticity roles were MAP2 and growth-associated protein-43 (GAP-43) [18]. Conversely to what was expected, GAP-43 was not elevated, as such an increase could have indicated a possible plasticity event occurring; this lack of response was possibly due to a lack of sensitivity of the measurement method used in the study [18]. Other studies, also looking at the effect of local stress due to tumoural growth, have focused on the hippocampus and its remodelling under stress [7,16,19,21,34]. A particular study performed on rat hippocampal tissue found that the glycoprotein M6a was expressed in the axons and dendrites of hippocampal neurons. The authors were able to demonstrate that overexpression and knockdown of M6a glycoprotein affects both neurite outgrowth and the density of neural spines in the hippocampal formation [16]. The study suggested that M6a may play an important role in synapse formation and, therefore, participate in the morphological modifications that occur in the hippocampi of brains under constant stress [16].

It is important to acknowledge that the analysis of the microscopic mechanisms governing neuronal structural changes was constrained by the limited volume of relevant literature. The majority of available studies focused on animal models, with relatively few investigations conducted using human subjects. Consequently, the understanding of microscopical molecular alterations induced by brain tumours remains a vast and underexplored area of research. As molecular techniques continue to advance, future studies are expected to increasingly elucidate the microscopic underpinnings of neuroplasticity in patients harbouring brain tumours. In this work, we aim to demonstrate neuroplastic events across multiple levels of organisation. We now proceed to review the macroscopic domain.

## 3. Bridging Molecular and Macroscopic Plasticity

**Figure 2:**
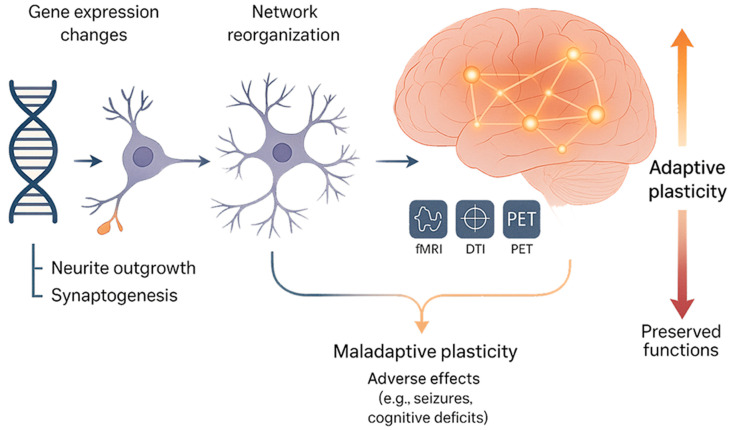
The mechanisms connecting microscopic and macroscopic neuroplasticity.

The transition from molecular alterations to macroscopic functional reorganisation represents a defining feature of neuroplasticity in the context of brain tumours. This complex process encompasses a wide spectrum of events, beginning with molecular and cellular perturbations and culminating in large-scale adaptations of functional networks (Figure 2). At the molecular level, diverse mechanisms are activated, including alterations in gene expression profiles, the modulation of neurotrophic factors, the reconfiguration of synaptic elements, and structural remodelling of neuronal and glial cells [16,17,18,19,20,21,22,23,24,25,26,27,28,29,30,31,32,33,34]. These foundational changes are not isolated occurrences; rather, they operate synergistically to drive the broader reorganisational processes essential for maintaining neural function in the face of progressive tumoural disruption.

For example, the upregulation of growth-associated proteins such as GAP-43 and M6a stimulates neurite outgrowth and synaptogenesis, thereby facilitating the establishment of new or alternative synaptic connections [16]. Similarly, molecules such as podoplanin, by interacting with signalling pathways involving p-Ezrin and CREB, contribute to enhanced neurogenesis and synaptic plasticity [19]. These molecular cascades are critical not only for the immediate structural reinforcement of injured regions, but also for enabling more extensive reorganisation across cortical and subcortical territories. At a higher level of integration, these microscopic changes manifest as macroscopic alterations in brain structure and function. Functional imaging studies, particularly using functional MRI (fMRI), diffusion tensor imaging (DTI), and positron emission tomography (PET), have demonstrated that regions adjacent to the tumour, or even anatomically distant areas, may exhibit functional hyperactivation, connectivity shifts, or recruitment of alternative pathways to sustain cognitive and motor functions [11,15,35,36]. In parallel, structural imaging modalities such as voxel-based morphometry (VBM) and surface-based morphometry (SBM) have significantly advanced our understanding of large-scale neuroanatomical adaptations in the tumoural brain. These techniques enable quantitative analysis of regional grey matter volume, cortical thickness, and surface area, providing complementary insights to functional imaging modalities [37]. Recent studies employing VBM have demonstrated structural plasticity not only in peri-tumoural regions, but also in distant contralesional areas, particularly within the frontal, temporal, and insular cortices. For instance, VBM analyses in patients with insular diffuse low-grade gliomas have revealed hypertrophy of the contralesional insula, interpreted as a compensatory structural reorganisation [38]. Similarly, SBM has been used to assess cortical thickness changes associated with tumour location and cognitive performance. Notably, increased cortical thickness in regions contralateral to the tumour has been interpreted as a marker of functional recruitment and reallocation of cognitive resources [34]. These findings underscore the existence of compensatory morphometric adaptations extending beyond the tumour core, reflecting the brain’s attempt to maintain network integrity and functional capacity despite progressive structural disruption. Moreover, these morphometric techniques hold promise for longitudinal monitoring of plastic changes and could aid in identifying biomarkers of neuroplastic capacity in individual patients.

Intraoperative mapping during awake craniotomy has further revealed that eloquent cortical areas can undergo substantial relocation or adaptation, challenging classical notions of fixed cortical localisation and underscoring their remarkable capacity for dynamic reorganisation [3,10,29]. Supporting these changes, astrocytic activation and angiogenesis play pivotal roles. Astrocytes, through the upregulation of proteins such GFAP, not only create a supportive microenvironment conducive to neuronal survival, but also actively participate in synaptic regulation and neurotransmitter homeostasis [25,31]. Concurrently, angiogenic processes ensure that newly recruited or reorganised neural circuits receive adequate metabolic support, facilitating sustained activity and plasticity [39].

Importantly, while molecular plasticity often performs a reparative or compensatory function, it can also give rise to maladaptive consequences. Aberrant synaptogenesis, disrupted inhibitory circuits, and glial scarring may contribute to network dysfunction, manifesting clinically as seizures, cognitive deficits, or behavioural disturbances [27,30,40]. Thus, the same molecular pathways that promote reorganisation can, under certain conditions, exacerbate pathology. Understanding this intricate and bidirectional relationship between molecular and macroscopic plasticity is of critical importance. It offers a conceptual framework for the development of targeted interventions aimed at amplifying beneficial plasticity while mitigating maladaptive processes. Advances in molecular biology, neuroimaging, and neuroengineering are poised to deepen our insight into this bridging process, paving the way for therapies that not only preserve, but actively enhance functional outcomes in patients harbouring brain tumours.

In summary, the journey from molecular alterations to macroscopic functional reorganisation encapsulates the essence of neuroplasticity in the tumoural brain. It is a dynamic, multi-layered process, simultaneously fragile and resilient, offering both challenges and opportunities for therapeutic innovation.

## 4. Plasticity Events at the Macroscopic Level

As mentioned previously, brain tumours greatly impact the dynamics of the organ’s physiology; they trigger genetic, proteinic, and molecular changes that are able to manifest on a larger scale (i.e., the macroscopic level). For many years, tumour surgeries in eloquent areas were considered high risk and, therefore, not feasible. However, this thinking was challenged by the emerging concept of neuroplasticity [40]. Brain plasticity was seen to occur after brain lesions (including surgery), where normal functions seemed to shift from lesioned areas to healthy ones [41]. When a tumour affects a part of the brain and its accompanying functions, other regions of the brain try to compensate for the functional deficit through plasticity. It is likely that many factors, such as the type and extent of the lesion, as well as the affected function, influence intrahemispheric or interhemispheric compensations; the exact mechanism for this process is not well understood yet [11,41,42].

In this section, we present how molecular neuroplasticity becomes visible at a macroscopic structural level in the brain; we divide the influence of its occurrence into spatial, temporal, and tumour size influences.

### 4.1. Macroscopic Plasticity Depending on the Spatial Location of the Tumour

#### 4.1.1. Cortical Plasticity

In 2016, Herbet et al. developed a map of the neuroplastic potential of a lesioned brain by generating a probabilistic atlas of functional plasticity from both anatomic magnetic resonance imaging results and intraoperative mapping data from two hundred and thirty-one patients who had undergone surgery for diffuse low-grade glioma (DLGG) [10]. They found that plasticity is higher at the cortex compared to connective tracts, where plasticity is rather low [10]. Similarly, another group described five patients with brain tumours in the primary motor cortex area, where only one of them showed significant functional deficits before treatment, possibly due to beneficial plastic events occurring in the other four that resulted in some retention of function [43]. Other neuroplastic events were depicted in a longitudinal study of twenty patients harbouring low-grade gliomas in the right cerebral hemisphere. In these patients, the presence of the tumours did not affect the results of neuropsychological tests when compared to the control group [44].

**Figure 3:**
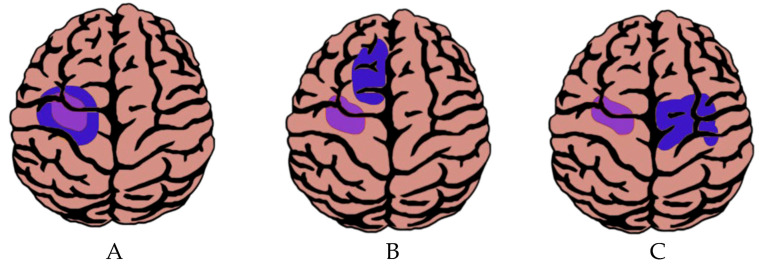
The figure shows neuroplasticity (in blue) induced by tumours (in purple), where (**A**) brain functions are retained within the tumour; (**B**) there is recruitment of peri-tumoural areas; and (**C**) there is recruitment of contralateral areas. The graphic is by Salvador Pineda and Estefano Cadena.

It is thought that the principal mechanism of cortical plasticity is functional reorganization. It occurs differently in many patients harbouring brain tumours and consists of the migration of functions through underlying networks [3,44]. Current research shows that cortical neuroplasticity follows a sequential model that begins intrinsically and progresses to remote areas; in other words, the neuroplastic mechanisms can occur initially with functions persisting within the tumour site, followed by the recruitment of ipsilateral areas, and ending in changes in brain areas far from the tumour on the contralateral side [3,29] (Figure 3).

In the first scenario (i.e., changes within the tumour site), the function can be conserved within the tumour area (Figure 3A). This phenomenon has been visualized by preoperative neuroimaging studies of patients with brain tumours and explains why some patients exhibit reduced or no functional alterations before surgery [2,43,44]. It is important to note that a lack of functional activity in functional neuroimaging results does not necessarily mean an actual absence of function in the brain tissue; there is a certain risk of misinterpretation due to tumour-induced neurovascular uncoupling [45].

In the second scenario, the compensation occurs in areas adjacent to the tumour (i.e., in the ipsilateral area) (Figure 3B). A study that illustrates this aspect reported that a patient without aphasia symptoms had a glioma in Broca’s area; his imaging study showed activation in the area inferior to the frontal cortex and in the right contralateral homologous area, which aided in preserving language functionality [46]. Another example was described by Fandino et al., where the premotor area along the superior frontal gyrus had compensatorily taken over certain motor functions in a patient with a slowly growing tumour in the central lobe [47]. These findings might be explained by processes previously described, such as (1) dormant areas becoming active; (2) compensatory areas being recruited as a result of the invading tumour; or (3) disinhibition of intraneuronal connections [5]. However, the plasticity around the tumour is not free from external influences that act on its growth, such as anti-tumourigenic, anti-angiogenic, and other treatments. Therefore, it is essential to understand both micro and macro mechanisms in neuroplasticity, since, for the patient to receive the utmost benefit, the greatest amount of brain matter must be viable [17].

In the third and final scenario, contralateral reorganization has also been described in the tumoural brain (Figure 3C). Studies have shown that neuronal hyperactivity occurs in the immediate vicinity to tumoural growth, as previously mentioned, but there is also evidence of reorganization and functional transfer to the contralateral hemisphere [42]. Additionally, for tumours located in the Rolandic region, several studies have reported activations within the contralesional primary motor cortex, the contralesional premotor area, and the contralesional supplementary motor area [11]. This type of cortical plasticity event has also been seen in brain areas such as the insula in the setting of diffuse low-grade gliomas (DLGG) [48]. Certainly, a study with 84 DLGG invading either the right or left insula found an increase in grey matter volume in the contralesional insula compared to healthy controls, as reported by voxel-based morphometry; these findings are a clear indicator of compensatory contralesional neuroplastic events [38]. Additionally, previous studies have shown that some patients with left hemispheric brain tumours have an increased propensity for developing right-sided language support to compensate for the tumour growth in the affected area. In an experiment by Fisicaro et al., the authors used direct cortical stimulation to study language function in 250 consecutive patients with gliomas [42]. High variability in language mapping within the dominant hemisphere was reported; specifically, the authors found out that sites normally associated with speech function were variably located along the cortex and extended well beyond the classic anatomical boundaries of the Broca and Wernicke areas [42]. Broca’s homologue in the right hemisphere was recruited after damage to Broca’s area proper in the typically dominant left hemisphere; this finding demonstrates that language function in this group of patients underwent plasticity to other areas of the brain not classically associated with language function after tumour invasion [42].

Beyond traditional eloquent cortices, recent studies have identified mesiotemporal structures, particularly the hippocampus, as key regions affected by tumour-related disruption and plastic reorganisation; certainly, patients with gliomas, even prior to any therapeutic intervention, often present with memory deficits, suggesting an early impact on mesiotemporal function [34,49]. However, neuroimaging studies have revealed compensatory hypertrophy or structural plasticity in contralesional hippocampi, indicating a potential adaptive response to preserve memory and other relevant cognitive functions [34,50]. These findings highlight the need to better characterise hippocampal plasticity in clinical practice, particularly given its critical role in memory and quality of life.

#### 4.1.2. Subcortical Plasticity

Subcortical plasticity refers to the capacity for functional reorganisation within the white matter pathways of the brain. Unlike cortical regions, which exhibit relatively high degrees of plastic adaptation, subcortical structures have traditionally been considered to possess limited neuroplastic potential. The neuroplastic potential map proposed by Herbet et al. systematically illustrated this distinction, indicating that associative tracts (such as the superior longitudinal fasciculus and arcuate fasciculus) and projection fibres (including the corticospinal tract and thalamocortical radiations) show markedly low tolerance to disruption, with limited evidence of compensatory remodelling after injury [10]. Their findings suggested that, in contrast to the dynamic reorganisation observed at the cortical level, subcortical connectivity tends to be more rigid, likely due to the highly structured and functionally specific nature of white matter pathways [10].

Nevertheless, emerging evidence suggests that subcortical plasticity, although considerably more constrained, does indeed occur under certain conditions. Clinical observations and neuroimaging studies have documented cases in which partial white matter tract damage, particularly in slowly growing lesions such as low-grade gliomas, has been associated with functional compensation and re-routing of information through adjacent or alternative pathways [41,51]. In these contexts, axonal sprouting, synaptogenesis at the grey–white matter junction, and reinforcement of redundant or less dominant pathways may contribute to partial functional recovery [41,51]. Several functional neuroimaging studies have also shown that the initial recruitment of contralesional cortical areas following unilateral brain lesions tends to diminish over time as more local networks regain function [9,52]. One plausible mechanism underlying this dynamic is the suppression of transcallosal inhibition, a phenomenon in which the affected hemisphere progressively reduces inhibitory signals transmitted via the corpus callosum to the homologous areas of the contralateral hemisphere, thereby promoting a more balanced interhemispheric interaction [9,51]. This process highlights a form of indirect subcortical plasticity involving interhemispheric fibres, with the corpus callosum acting as a critical modulator of plastic responses.

Although subcortical plasticity is less robust than cortical reorganisation, its clinical relevance is significant, particularly in the context of neurosurgical planning for intrinsic brain tumours. Preservation of critical subcortical pathways during surgery has been shown to correlate strongly with postoperative functional outcomes, reinforcing the need to account for the limits and possibilities of subcortical neuroplasticity [53]. Future advances in diffusion tractography, connectomics, and intraoperative mapping are poised to further elucidate the extent and mechanisms of white matter reorganisation.

### 4.2. Macroscopic Plasticity Depending on Temporal Aspects

It is important to emphasize that slow and acute lesions and tumours involve very different patterns of reorganization. The extent and speed of the growing tumour have a direct association with the amount of plasticity in the brain; for instance, research has showed that the recruitment of remote brain areas in the ipsi- and contra-lesional hemispheres is much more efficient in slow-growing tumours compared to acute lesions [11]. Evidence suggests that plasticity may be most effective when the injury is minor and slowly increases over time, as found in some observations of progressive neural degeneration. Slow-growing tumours may gradually upregulate compensatory events that play a key role in maintaining the homeostasis of the tumoural brain [17]. Slow tumour progression may be the key to cortex rewiring and functional adaptability; for instance, Hayashi et al. found out that the cortical motor function area shifted posteriorly and crossed the central sulcus after a slow-progressing glioma developed in the primary motor cortex [54]. This neuroplasticity was mainly attained thanks to brain reorganization facilitated by the tumour’s growth rate. Certainly, preservation of function after the development of a low-grade glioma and the timely reorganization and redistribution of function to unaffected regions is not an uncommon phenomenon; another study showed that brains affected by these types of tumours have a better recovery when preserving the subcortical pathways and reorganizing functions along alternative networks, achieving long-term recovery and the timely redistribution of functions to the preserved regions of the cortex [40].

### 4.3. Macroscopic Plasticity Depending on the Size of the Tumour

Finally, another influential factor of macroscopic plasticity is the size of the tumour, which has been shown to have an impact on the reorganization of the functional areas. While larger tumours could reasonably be expected to cause greater functional disruption, clinical evidence suggests a far more nuanced relationship. In particular, tumour cell density, rather than size per se, appears to exert a more decisive influence on the extent of functional impairment and the brain’s capacity for adaptive reorganisation [3,10,40]. High cellularity often induces more abrupt, infiltrative disruptions of functional circuits, thereby limiting the brain’s ability to engage compensatory mechanisms. By contrast, low-density, slow-growing tumours tend to permit surrounding neural structures sufficient time to undergo progressive, plastic adaptations, thereby preserving function despite the increasing tumour burden [3,10,40]. Importantly, tumour size alone does not necessarily predict the clinical outcome. Several studies have demonstrated that patients harbouring large low-grade gliomas (i.e., low-density tumours, in general) can experience minimal or even no major neurological deficits, even following extensive surgical resections [3,11,55]. This striking phenomenon highlights the extraordinary plastic potential of the human brain, particularly when tumour growth is indolent, allowing gradual reallocation of function to adjacent or even contralateral regions [11]. Certainly, low-grade gliomas, characterised by their slow expansion and lower proliferative indices, provide the clearest clinical model for this dynamic. Their chronic progression serves as a continuous stimulus for neuroplastic mechanisms, fostering synaptic reorganisation, remodelling of white matter tracts, and recruitment of compensatory or parallel circuits [29,56]. At the microscopic level, molecular pathways promoting axonal sprouting, dendritic remodelling, and astroglial support are persistently engaged, while at the macroscopic level, functional networks progressively reshape themselves to maintain cognitive and motor capacities.

The interplay between slow tumour progression and high intrinsic plasticity permits aggressive surgical strategies aimed at maximal safe resection, often guided by intraoperative awake mapping and continuous functional monitoring [29,51,57]. Such approaches challenge the classical localizationist view of rigid cortical specialisation, instead supporting a dynamic and distributed model of brain function that is highly responsive to chronic pathological stress. These findings underscore the critical importance of considering tumour dynamics, cellular characteristics, and the microenvironment, rather than relying solely on size, when evaluating prognosis and planning intervention. They also reaffirm the brain’s remarkable, though not limitless, capacity for adaptive reorganisation in the setting of disease.

## 5. Clinical Relevance of Neuroplasticity in Gliomas

Understanding neuroplasticity has revolutionised neurosurgical approaches to gliomas. Historically, tumours located within eloquent regions (i.e., motor, language, and attentional cortices) were considered inoperable due to the high risk of severe functional impairment; surgical interventions were often limited to diagnostic biopsies or partial resections to minimise morbidity. However, accumulating evidence has demonstrated that neuroplasticity enables the functional remodelling of critical brain regions, allowing for more extensive resections while preserving or even improving patients’ quality of life. Duffau et al. demonstrated that up to 95% of patients with low-grade gliomas involving eloquent cortices could undergo extensive resections with preserved or only transient neurological deficits when intraoperative electrical stimulation mapping was employed [5]. Their work highlighted the critical importance of real-time functional identification during surgery, enabling the surgeon to tailor resections to the patient’s evolving anatomo-functional architecture. Similarly, other groups have reported that among patients with tumours involving the primary motor cortex, approximately 80% achieved postoperative functional recovery [47]. Perioperative functional MRI revealed compensatory reorganisation of motor pathways, with the recruitment of perilesional and contralateral regions supporting recovery.

Plasticity, however, is not confined to motor functions; a longitudinal study involving 20 patients with right hemisphere low-grade gliomas revealed that attentional performance, as assessed through serial neuropsychological testing, remained stable over time despite tumour progression [44]. This stability was attributed to compensatory functional recruitment of unaffected networks, supporting the brain’s remarkable capacity to reorganise attentional systems even in the presence of a growing mass lesion. Moreover, functional recovery extends robustly to language systems. One study reported that approximately 38% of patients with left hemispheric gliomas showed recruitment of right hemisphere homologues of Broca’s and Wernicke’s areas, as demonstrated by direct cortical stimulation and functional imaging [42]. This interhemispheric shift suggests that, particularly in slow-growing tumours, language functions are not fixed but can migrate to alternative cortical territories, a finding of immense clinical relevance. These findings have profound implications for patient management. Certainly, early surgical intervention, particularly when guided by advanced preoperative functional imaging (e.g., fMRI, DTI tractography) and intraoperative mapping, has been associated with longer progression-free survival and overall survival in glioma patients [58,59]. The ability to predict and monitor neuroplastic responses allows clinicians to individualise surgical strategies, maximise the extent of resection, and minimise the risk of postoperative neurological morbidity. Consequently, neurosurgical philosophy has shifted from a cautious, symptom-preserving approach to an aggressive yet functionally informed strategy aimed at optimising both oncological and functional outcomes, lowering the surgical footprint in the brain as much as possible.

In summary, the integration of neuroplasticity concepts into clinical practice has profoundly transformed the paradigm of glioma management. The brain’s dynamic capacity for reorganisation offers a powerful ally, enabling clinicians to push the boundaries of tumour resection while safeguarding patients’ cognitive and functional status.

## 6. Limitations and Future Directions

Despite considerable advances in the understanding of neuroplasticity in the context of brain tumours, several important limitations persist. A substantial proportion of molecular evidence is derived from preclinical studies, primarily rodent glioma models, which, while informative, cannot fully replicate the complexity of the human brain microenvironment. Consequently, the extrapolation of these findings to clinical settings must be approached with caution. Longitudinal human studies remain scarce; most available data are cross-sectional, providing only static insights into plasticity rather than capturing its dynamic and evolving nature across the course of tumour progression and treatment. This limitation restricts our ability to understand the temporal unfolding of neuroplastic mechanisms. Functional imaging modalities, although powerful, are not without technical constraints. Neurovascular uncoupling in peri-tumoural regions may result in misleading functional MRI signals, complicating the interpretation of true reorganisation. Furthermore, variability in individual plastic potential, driven by factors such as age, tumour histology, growth kinetics, genetic background, and comorbidities, adds another layer of complexity, making the prediction of functional outcomes on a case-by-case basis inherently difficult.

Future research must address these gaps. Priority areas include the development of reliable biomarkers to predict neuroplastic capacity; the integration of molecular imaging and connectomics to achieve higher-resolution mapping of reorganisation; longitudinal, multicentric studies combining functional imaging, electrophysiology, and clinical outcomes; and the exploration of pharmacological or neuromodulatory interventions aimed at enhancing beneficial plasticity. Furthermore, given the growing recognition of mesiotemporal plasticity, particularly in the hippocampus, future work should also explore the therapeutic modulation of these processes. Cognitive rehabilitation interventions designed to stimulate hippocampal-dependent memory circuits may offer promising avenues for preserving or restoring cognitive function, as emerging evidence suggests that structured cognitive training and neurofeedback could leverage endogenous plastic mechanisms, especially in patients with slow-growing lesions [60,61,62,63]. Tailored rehabilitation strategies that promote mesiotemporal plasticity should be considered in the context of multimodal treatment planning. Ultimately, a deeper understanding of the molecular and network-level mechanisms governing neuroplasticity will be essential for the design of personalised interventions that optimise functional recovery and improve oncological outcomes.

## 7. Conclusions

Understanding the molecular underpinnings of neuroplasticity in brain tumours has reshaped the clinical approach to diagnosis, prognosis, and surgical intervention. The evidence reviewed here highlights the fact that genetic, protein-level, synaptic, and structural changes constitute a coordinated response that enables functional reorganisation at a macroscopic scale. This plasticity not only permits the preservation of motor, language, and cognitive functions despite progressive tumoural insult, but also paves the way for more aggressive yet safer resections. However, molecular and imaging studies remain predominantly preclinical, underscoring the need for longitudinal, multimodal human investigations. Future efforts to refine the modulation of neuroplastic pathways may ultimately improve both oncological outcomes and functional recovery, reinforcing the brain’s remarkable yet complex capacity for adaptation in the face of disease.

## Figures and Tables

**Table 1:** The main molecules and proteins involved in neuroplasticity in the setting of tumours or processes related to tumours.

Molecule/ Protein	Setting *	Category ^¶^	Regulation	Plasticity Change	Promotes (+) Reduces (−) Plasticity	Reference
M6a	stress	5	↑	neurite and filopodium growth + synapse formation	(+)	[16]
MAP2	gliosarcoma	5	↑	neuronal migration and neurite outgrowth	(+)	[17,18]
GFAP	gliosarcoma	6	↑	synaptic plasticity	(+)	[17]
vWF	gliosarcoma	7	↑	angiogenesis	(+)	[17]
Synaptophysin	astroglioma medium	6	↑	synaptogenesis	(+)	[16,20]
MHC I	astroglioma medium	6	↑	negative influence on synaptic stability	(−)	[20]
Glutamate	glioma	6	↑	impairment of excitatory and inhibitory synaptic transmission	(−)	[21]
Glu/GABA	glioma	6	↑	impairment of excitatory and inhibitory synaptic transmission	(−)	[21]
Neurofilament	glioblastoma	6	↓	staining showed gradual reduction within tumour centre	(−)	[23]
Tyrosine hydroxylase	glioblastoma	6	↓	staining showed gradual reduction within tumour centre	(−)	[23]
Dopamine transporter	glioblastoma	6	↓	decrease in density of dopaminergic endings	(−)	[23]
GAP-43	diffuse astrocytoma	5	No change	no change in tumour model vs. control	No change	[18]
Podoplanin	overexpression experiments	7	↑	brain neuronal outgrowth, synaptic plasticity	(+)	[19]

* In rodent models. ^¶^ Categories are based on the classification suggested by Keyvani & Schallert in 2002 [1]; category 5 involves neuronal growth-associated molecules and cytoskeletal molecules, category 6 involves synapse-related molecules, and category 7 involves adhesion molecules. Arrows indicate an increase or decrease in the regulation of the molecule and/or protein.
